# Supplementary material for: Learning curve and surgical outcome of robotic assisted colorectal surgery with ERAS program
Source: Sci Rep. 2022 Nov 29;12:20566. doi: 10.1038/s41598-022-24665-w (PMC9709162; doi:10.1038/s41598-022-24665-w)
Supplement: Supplementary file 1 — Supplementary Information 1. [file 41598_2022_24665_MOESM1_ESM.zip › LSC Raw data-Ñ[▒K/121/H_ñw╝╨░O▒Kñσ.pdf]

正式摘要(電子簽章：00000213057102)

W55 / W55 ☐英文標題(注意:無英文姓名-請補病人基本檔!) ☐【讀取】憑證

臺中榮民總醫院

出院病歷摘要

轉入號 : 轉出號 :

[redacted]

(8)流水編號	
(9)入院日期	110/03/07 (2021) 胃腸肝膽科 W55 - 273
(10)轉科日期	110/03/15 (2021) - 直腸外科 - W55-273
(11)出院日期	110/03/25 (2021) 住院天數計 18 日

診斷

- 入院 : 1. multiple liver masses
  [Hx]
  1. Hypertension for years under norvasc and bokey;
  2. Insomnia under xanax;
  3. External hemorrhoid
- 出院 : 1. Sigmoid colon cancer,
  1. Sigmoid colon cancer, pT3N2a, Adenocarcinoma, muliple liver and LN metastasis, pending gene test

主訴

- Intermittent upper abdominal fullness, especially right upper area, body weight loss for 8 kgs within 3 months.

病史

- This 59-year-old woman with history of (1) Hypertension for years under norvasc and bokey; (2) Insomnia under xanax; (3) External hemorrhoid. Her ADLs is total dependent. This time she had intermittent upper abdominal fullness, especially right upper area, body weight loss for 8 kgs within 3 months. She visited local clinic 9 days ago where multiple liver mass were found by sonography, then referred to our hospital for help. Abdominal CT revealed multiple liver tumors, rule out cholangiocarcinoma or liver metastasis. Normal AFP and Ca-199. Negative of hepatitis marcker, but elevation of CEA(102.6ng/ml) found at our GI OPD. Admission for liver biopsy and tumor survey. Besides, bowel habit change with mucinous stool, sometimes tinged bloody noticed within one year.
  At our emergent department where initial vital sign showed BP:155/72 mmHg, RR:18 bpm, PR:89 bpm, BT:35.5℃. Physical examination showed alert and oriented consciousness, smooth respiratory pattern, soft abdomen with nomoactive bowel sounds, mild A1 tenderness, bilateral leg pitting edema:trace. Laboratory data showed anemia(Hb:7.7g/dL) and iron deficiency. Under the tentative diagnosed of hepatic tumors she was admitted for further management and treatment.

體檢發現

- drug: bokey (3/2) ; xanax norvasc
  - General appearance: A well-nourished person, with acute ill looking. Consciousness: Alert Pupil size: 2/2 mm Light reflex:
    - Normal light reflex

    Vital signs:

date	體溫(BT)	脈搏(P)	呼吸(R)	血壓(BP)	身高(BH)	體重(BW)	BMI
20210307	36℃	88次/分	17次/分	158/72mmHg	158cm	48.6kg	19.5
  - Skin
    - Normal skin turgor , No jaundice , No cyanosis , No rash , No wound / ulcer / scar
  - Lymphadenopathy
    - No lymphadenopathy
  - HEENT
    - Head: No shape deformity , No hair change , Normal lip
    - Eyes: *Conjunctiva: Normal , *Sclera: Not icteric , *Cornea: Clear , *

- Eyelids: Normal
    - Ears: No deformity
    - Nose: Normal appearance
    - Mouth and throat: No oral mucosa change , No throat injection , No tonsil enlargement
- Neck
    - No thyroid enlargement , Neck: Jugular vein
- Chest
    - Inspection: Normal and symmetric respiratory movement , No deformity , Normal breast
    - Palpation: No palpable mass , No tender point
    - Auscultation: No rales , No rhonchi , No wheezing , Chest / Auscultation / Stridor No stridor , No peripheral brochial sound , No friction rub
- Heart
    - Inspection: No visible apical impulse
    - Palpation: Not palpable PMI , No thrill , No heave
    - Auscultation: Regular heart beats , No S3 , No S4 , No murmur
- Abdomen
    - Inspection: No distension , No striae , No caput medusae , No hernia , No Grey-Turner's sign , No Cullen's sign , No superficial vein engorgement
    - Auscultation: Normal bowel sound ,
    - Palpation: No hepatomegaly , location: A1 , No rebounding pain , No palpable mass , No splenomegaly
    - Percussion: No shifting dullness , No central tympanic
- Genito-anus patient refuse
- Back and spine
    - No deformity , No knocking pain
- Extremities
    - No deformity , No clubbing finger , No pitting edema , Normal joint , Normal peripheral pulse
    - No palm erythema , No muscular tenderness
- Neurological examination
    - No mental status change
    - Normal cranial nerve
    - Normal meninges
    - Normal motor
    - Normal sensory
    - Normal cerebellum
    - Normal Stance & Gait

手術日期及方法

- 手術日期及方法20210315 1. Laparoscopic anterior resection
  2. Intraoperative colonoscopy : (-)
  3. High ligation (+) : At IMA root
  4. EndoGIA (60-4.1) X1 + EEA (29 mm) were used
  5. Proximal cut end : X cm, distal cut end : X cm
  6. One 12 Fr. penrose drainage tube was placed into the pelvic cavity from the LLQ of the abdomen
  7. ADHESION BARRIER FILM was placed below the central abdominal wound.
  8. The colon was not taken down from the splenic flexure site
  9. The injured middle T-colon was repaired with Gambee Suture via 3-O Vicryl, then the outer layer was re-inforced by interrupted 3-0 silk Lembert sutures.

住院治療經過

- After admission, GI arranged liver biopsy for tumor survey. The pathology report showed metastatic adenocarcinoma. Painless EGD and colonscope for tumor survey were done on 3/11 and L-scope revealed R/I sigmoid colon cancer with easy touch bleeding and lumen obstruction. CRS was consulted and Laparoscopic Anterior resection and anastomosis was arranged on 3/15. After transfer to CRS service, Lap LAR with ileostomy was performed smoothly on 03/15. She tried water on 03/16 and BLAND I on 03/18. Flatus and stool passage were also noted on 03/18. She also tolerated well with BLAND III on 03/19. Surgical wound was clean and no discharge. Penrose content was light yellowish and no infectious signs were noted. However, low serum

- albumin and bilateral lower legs edema were noted. Albumin supplement with Rosis use were applied. Edema was then improved. Penrose was removed on 03/22. Stitches and CVC were removed on 03/23. Bilateral limbs also improved after application of rosis and spirolactone. Since the patient was under a relatively stable codition, she was then discharged on 03/25. OPD f/u was also arranged.

合併症

一般檢查

- 累積報告：

DATE	TIME	ALB	TP	BIL.T	BIL.D	ALKP	AST	ALT	LDH	CHOL	TG	HDL-C	LDL	UA	NH3	AMY
1100315	0915			0.6	0.2	305	76	27								
1100315	2009	2.6		1.11	0.33		77	29								
1100317	0852	3.0	5.9	0.67	0.27	193	163	67	2260							
1100319	0850	3.0	5.7	0.79	0.27	190	54	30	1795							
1100322	0943	3.8	6.6	0.99	0.35	343	57	26	1902							
1100325	0904	3.3		0.9				32								

DATE	TIME	WBC	RBC	HGB	HCT	MCV	PLT	BLST	NEUT	BAND	SEG	LYM	MON	EOS	BAS
1100307	0946			7.7											
1100308	1932			6.2											
1100309	0830			8.3											
1100315	0939	7350		9.6		77.3	489		68.0			22.3	7.5	1.4	0.8
1100315	2007	2730		9.4	30.7		335		80.9			16.5	1.5	0.7	0.4
1100317	0902	15930	3.92	9.9	30.6	78.1	345				90.0	8.0	2.0	0.0	0.0
1100319	0938	9890	3.72	9.5	29.8	80.1	314		84.5			9.5	5.2	0.7	0.1
1100322	0920	12020	4.15	10.4	33.3	80.2	450								
1100325	0916	9090	3.98	9.9			552		77.6			14.6	6.8	0.7	0.3

DATE	TIME	NA	K	CL	CA	BUN	CREAT	Mg	P	CRP	ESR	PCT	LIPASE	LACTATE
1100307	0947	133	3.6		8.1	10	0.70							
1100315	0915	136	3.4		8.8	6	0.63							
1100315	2009	135	3.3		8.1	6	0.63							
1100317	0753													13.2
1100317	0852	134	4.1	98	7.8	22	1.76							
1100319	0850	131	3.3	96	7.3	17	0.62							
1100322	0943	132	3.3	88	8.2	11	0.61							
1100325	0904	131	3.2		8.1	8	0.62							

DATE	TIME	GLUCOSE	PAB	TRF	ZN	CU	KETONE	CK	CKMB	PT-P	PT-C	APTT-P	APTT-C	TRO-T
1100307	0947	110						251						
1100315	0923									11.4	10.6	28.1		

特殊檢查

病理報告

- 活體組織切片病理報告　手術日期：**110/03/08**　病理號：**S1007404**

診斷醫師:詹以吉 報告日期:110/03/11
Pathologic diagnosis:
Liver, biopsy --- Metastatic adenocarcinoma, moderately differentiated, consistent with colorectal origin.
Ancillary study for diagnosis:
1. PAS, reticulum, Masson's trichrome stains done.
2. IHC stains for SATB2 (+), CDX2 (+) and CK7 (-) done.
Gross description:
The specimen consists of multiple strips of tan soft tissue, up to 0.5x0.1x0.1 cm. All for section.
Microscopic description:
Section shows liver tissue with tumor composed of proliferative atypical colorectal type glands with luminal dirty necrosis. Tumor cells have enlarged nuclei, increased nucleo-cytoplasmic ratio and prominent nucleoli.
Note:
The case has been peer reviewed by two doctors and the diagnosis is concurred in intradepartment consensus meeting on 2021/03/11.
#T-62000_2 #M-81406_2 2 1143 00CM0F

- 活體組織切片病理報告 手術日期：**110/03/11** 病理號：**S1007977**

診斷醫師:孫志豪 報告日期:110/03/15
Pathologic diagnosis:
Stomach, antrum, endoscopic biopsy --- Chronic active gastritis with intestinal metaplasia.
Ancillary study for diagnosis:
Giemsa stain for Helicobacter Pylori identification done.
Prognostic and predictive factor:
Helicobacter-like microorganisms (+) found.
Gross description:
The specimen consists of a piece of tan soft tissue, 0.4x0.3x0.3 cm.
All for section.
Microscopic description:
1. Neutrophils activity- Moderate.
2. Chronic inflammation/Mononuclear cells infiltration- Moderate.
3. Atrophy of glands- Cannot be well-evaluated.
4. Intestinal metaplasia- Mild.
#T-57000_2 #M-43000_2 0 1340 00HP00
#T-57000_2 #M-73320_2 0 1340 000000

活體組織切片病理報告 手術日期：**110/03/15** 病理號：**S1008410**

診斷醫師:詹以吉 報告日期:110/03/22
Pathologic diagnosis:
1. Sigmoid colon, laparoscopic anterior resection ---
Adenocarcinoma, moderately differentiated. Tumor invaded to mesocolic soft tissue and metastasized to mesocolic lymph nodes (4/25). The bilateral cut ends are free of tumor.
2. Colon, cut end, distal, resection --- Unremarkable.
3. IMA root, dissection --- Unremarkable (0/4).
Ancillary study for diagnosis:
IHC stains for MLH1, MSH2, PMS2 and MSH6 are done on section H. All above markers are preserved.
Prognostic and predictive factor:
1. Tumor size: 6x4x1 cm.
2. Depth of invasion: Pericolorectal tissue.
3. Histologic grade: G2: Moderately differentiated.
4. Angiolymphatic invasion: Not identified.
5. Perineural invasion: Not identified.
6. Tumor deposits (discontinuous extramural extension): Not identified.
7. Macroscopic tumor perforation: Not identified.
8. Surgical Margins:
a) Proximal Margin: Uninvolved by invasive carcinoma, distance of tumor from margin: 3 cm.
b) Distal Margin: Uninvolved by invasive carcinoma, distance of tumor from margin: 2.2 cm.
c) Radial or Mesenteric Margin: Uninvolved by invasive carcinoma, distance of tumor from margin: More than 1 cm.
9. Lymph node status: Involved (4/29).
10. Extranodal involvement: Present.
11. Tumor regression grading S/P CCRT: No known presurgical therapy.
12. Type of polyp in which invasive carcinoma arose: Not identified.
13. Additional pathologic findings: Not identified.
14. Pathological TNM Stage: pT3N2a(According to the eighth edition, American Joint Committee on Cancer Staging Guidelines for Tumors).
15. TNM descriptors: Not applicable.
Gross description:
The specimen consists of 1) a segment of colon, 11 cm in length and 4 cm in proximal cut end circumference, 4 cm in distal circumference, and mesocolic soft tissue. A gray tan firm annular elevated and centrally ulcerative tumor, 6x4x1 cm, is noted, 2.2 cm to the nearest cut end. On cross section, the tumor invades to the mesocolic soft tissue with more than 10 mm to the radial margin, and serosal retraction is also noted. The both cut ends are free of tumor grossly. 2) distal cut end, a piece of tan mucosa-covered soft tissue, 1.5x1.5x0.5 cm. 3) IMA root, a piece of tan yellow soft

- tissue, 3x2x0.5 cm. All for section: A) specimen 2 B) specimen 3; Representative part for section: C) distal cut end D) proximal cut end E-H) tumor and serosa J) CFM K-P) LN.
  Note:
  1. The interpretation of MMR panel and recommended studies are as below:
  (1) No loss of nuclear expression of MMR proteins: low probability of microsatellite instability-high (MSI-H).
  (2) Loss of nuclear expression of MLH1 and PMS2: testing for methylation of the MLH1 promoter and/or mutation of BRAF is indicated (the presence of a BRAF V600E mutation and/or MLH1 methylation suggests that the tumor is sporadic and germline evaluation is probably not indicated; absence of both MLH1 methylation and of BRAF V600E mutation suggests the possibility of Lynch syndrome, and sequencing and/or large deletion/duplication testing of germline MLH1 may be indicated).
  (3) Loss of nuclear expression of MSH2 and MSH6: high probability of Lynch syndrome (sequencing and/or large deletion/duplication testing of germline MSH2 may be indicated, and, if negative, sequencing and/or large deletion/duplication testing of germline MSH6 may be indicated).
  (4) Loss of nuclear expression of MSH6 only: high probability of Lynch syndrome (sequencing and/or large deletion/duplication testing of germline MSH6 may be indicated).
  (5) Loss of nuclear expression of PMS2 only: high probability of Lynch syndrome (sequencing and/or large deletion/duplication testing of germline PMS2 may be indicated).
  (6) Please note: There are exceptions to the above IHC interpretations. These results should not be considered in isolation, and clinical correlation with genetic counseling is recommended to assess the need for germline testing.
  2. This case has been peer reviewed by two doctors.
  #T-59300_2 #M-81403_2 2 1105 S0000F
  #T-C4560_2 #M-81406_2 2 1105 00000F

其它

出院時情況

- 3.改門診治療

出院指示

藥名	劑量	劑量單位	用法	頻次	數量	數量單位	現況	天數	單位
Iodosorb powd. 3Gm	1	PKG	TOP	QD	3	PKG	TH	3	天
Norvasc tab 5mg	1	TAB	PO	QD	7	TAB	TH	7	天
$Lederscon tab	1	TAB	PO	QID	28	TAB	TH	7	天
Through tab	1	TAB	PO	QNPRN	7	TAB	TH	7	天
Spironolactone-25	1	TAB	PO	BID	14	TAB	TH	7	天
MOSAD tab 5mg	1	TAB	PO	TID	21	TAB	TH	7	天
(□)Rosis tab 40mg	1	TAB	PO	BID	14	TAB	TH	7	天
Xanax tab 0.5mg	1	TAB	PO	HSPRN	7	TAB	TH	7	天

出院計劃

主治醫師 2062F 蔣鋒帆 / 住院醫師 9757K 翁明謙

正式摘要(電子簽章：000000213057102)

W55 / W55

doctorRank:B
